# SARS-CoV-2 in mink farms in British Columbia, Canada: A report of two outbreaks in 2020–2021

**DOI:** 10.14745/ccdr.v48i06a05

**Published:** 2022-06-09

**Authors:** Adrianna Paiero, Emily Newhouse, Elaine Chan, Veronic Clair, Shannon Russell, James Zlonsnik, Natalie Prystajecky, Erin Fraser

**Affiliations:** 1 Fraser Health Authority, Surrey, BC; 2 University of British Columbia, Vancouver, BC; 3 British Columbia Centre for Disease Control, Vancouver, BC; 4 Canadian Field Epidemiology Program, Centre for Emergency Preparedness and Response, Public Health Agency of Canada, Ottawa, ON

**Keywords:** mink, spillover, zoonotic disease, SARS-CoV-2, COVID-19, One Health

## Abstract

**Background:**

Since April 2020, mink have been recognized as a potential reservoir for severe acute respiratory syndrome coronavirus 2 (SARS-CoV-2) and a potential source of new variants. The objective of this report is to describe the epidemiological investigation and public health response to two coronavirus disease 2019 (COVID-19) outbreaks that involved both humans and farmed mink.

**Methods:**

An outbreak was declared on December 4, 2020, following detection of two COVID-19-positive farmworkers and elevated mink mortality on a mink farm (Farm 1) in British Columbia. The second cluster was detected on Farm 3 following detection of 1) a COVID-19 case among farm staff on April 2, 2021, 2) an indeterminate result from farm staff on May 11, 2021, and 3) subsequent SARS-CoV-2-positive mink in May 2021. Quarantine of infected farms, isolation of workers and their close contacts, and introduction of enhanced infection control practises were implemented to break chains of transmission.

**Results:**

Among mink farmworkers, 11 cases were identified at Farm 1 and 6 cases were identified at Farm 3. On both Farm 1 and Farm 3, characteristic COVID-19 symptoms were present in farm employees before signs were observed in the minks. The viral sequences from mink and human samples demonstrated close genetic relation. Phylogenetic analyses identified mink intermediates linking human cases, suggesting anthropo-zoonotic transmission.

**Conclusion:**

These were the first COVID-19 outbreaks that included infected mink herds in Canada and identified potential anthropogenic and zoonotic transmission of SARS-CoV-2. We provide insight into the positive impact of regulatory control measures and surveillance to reduce the spillover of SARS-CoV-2 mink variants into the general population.

## Introduction

Mink—a semiaquatic, carnivorous mammal of the genus Neogale—have been identified as a potential reservoir of severe acute respiratory syndrome coronavirus 2 (SARS-CoV-2), the virus that causes coronavirus disease 2019 (COVID-19) ((1)). Since April 2020, twelve countries have reported SARS-CoV-2 infection in mink ((2,3)). Genetic analyses of outbreaks in Denmark and the Netherlands suggest potential anthropo-zoonotic origins and spillover of mink-divergent SARS-CoV-2 into the greater community, highlighting a biosecurity risk resulting from genetic diversification following adaptation of the virus in a new host ((4–7)).

The Fraser Valley contains all mink farms in British Columbia (BC), Canada, and produced 23% of Canadian mink pelts in 2020 ((8)). BC’s farms are located close to major population centres. The historical herd size is significantly smaller than Danish and Dutch farms, with less reliance on centralized infrastructure for feeding and pelting. Since the beginning of the pandemic, three BC mink farms have experienced SARS-CoV-2 transmission within mink herds, with two of these outbreaks (Farm 1, Farm 3) involving documented human cases ((9)).

We report on the epidemiological investigation of the two outbreaks that involved genetically linked human and mink infections with SARS-CoV-2 occurring between December 2020 and November 2021 in BC. We reflect on the impact of control measures, vaccines and active surveillance, highlighting how these interventions may have created the unique epidemiological profile observed on Farm 3 and provide comparisons to Farm 1 and other outbreaks described in the literature.

## Methods

### Overview

The outbreak on Farm 1 was detected on December 2, 2020, during the pelt-harvesting season after two farmworkers tested COVID-19-positive at a community testing site. The farm owner identified an increased overall mortality rate of approximately 1.5% in the herd of 15,000 mink in the preceding week. A herd veterinarian was deployed to sample mink mortalities on the farm. On December 4, 2020, Public Health (PH) declared an outbreak in mink and farmworkers and the Ministry of Agriculture, Food and Fisheries (MAFF) quarantined the farm after four of five mink samples tested non-negative. On February 24, 2021, the outbreak was declared over.

On April 2, 2021, a farmworker on Farm 3 tested positive prior to achieving vaccine-mediated immunity through the mink farmworker surveillance system established in February 2021 ((9)). On May 11, 2021, an indeterminate result from another worker and SARS-CoV-2-positive mink mortalities were identified. On May 12, 2021, MAFF placed the farm under quarantine and an outbreak investigation began and was ongoing as of November 1, 2021. For both farms, the criteria for an outbreak to be declared over was for the farm to have neither positive nor indeterminate human or mink samples detected for two consecutive 14-day incubation periods.

### Laboratory investigation

The British Columbia Centre for Disease Control (BCCDC) Public Health Laboratory conducted real-time polymerase chain reaction-based (RT-PCR) SARS-CoV-2 testing using RdRP and E gene targets; specimens were confirmed SARS-CoV-2 positive at a cycle threshold (Ct) value ≤35. The MAFF performed preliminary animal testing (reported as negative or non-negative based on RT-PCR of the E gene target) that was confirmed with similar assays validated in animals at the National Centre for Foreign Animal Disease laboratory in Winnipeg, Canada.

All human and animal samples with a PCR-confirmed positive test underwent next-generation whole genome sequencing (WGS) using laboratory methods described in detail elsewhere ((10)). Briefly, samples were sequenced on an Illumina MiSeq or NextSeq instrument using a tiled 1,200 bp amplicon scheme and analyzed using a modified ARCTIC Nextflow pipeline ((10)). Sequences passing quality control (85% genome completeness, 10X depth of coverage and no quality flags) were included in the phylogenetic analysis. Phylogenetic trees were constructed using Nextstrain ((11)) and samples were manually assigned to a genetic clade based on an inclusion criterion of three mutations or fewer. According to our clade calling scheme, 0 mutations were “identical”, 1–2 mutations were “nearly identical”, 3 mutations were “similar” and more than 3 mutations were “different”. This scheme aligns with the previously reported mutation rate in humans of approximately one mutation per two-week period ((12)). Samples were assigned a sub-clade designation (e.g. Clade 1.1) to denote clusters of genetically identical sequences. Lineage assignment was performed using the Phylogenetic Assignment of Named Global Outbreak Lineages tool (PANGOLIN) Version V.3.1.17 ((13)).

### Case finding and investigation

For these investigations, the case definitions were as follows:

- Confirmed case: an individual who worked on the farm **and** had a positive RT-PCR (Ct:≤35)
- Epidemiologically linked case: an individual who worked on the farm **and** had an indeterminate RT-PCR (Ct:36–50) **and** reported respiratory symptoms consistent with SARS-CoV-2 in the two weeks prior to testing **or** was a household contact of a confirmed case

Public Health conducted investigations of confirmed cases within 24 hours of notification. Interviews explored illness onset date, symptoms, exposure history, risk factors, close contacts and connections to other mink farms. Owners of infected farms confirmed each case’s duties and identified farm contacts. Cases were instructed to isolate for 10 days. Close contacts of SARS-CoV-2-positive mink and humans were advised to self-isolate for 14 days since their last exposure and seek testing if symptoms developed.

### Animal and wildlife investigation

Herd veterinarians conducted weekly animal sampling on site. Animal sampling included nasopharyngeal swabbing of mink mortalities and live mink. Wildlife captured by hunters and trappers in the less than 2 km perimeter of each premise provided insight into the spillover from escaped mink into the surrounding wildlife, as described in Strang *et al.* ((14)). No wildlife samples tested positive for SARS-CoV-2.

### Epidemiological and statistical analyses

Case and contact management details were available through PARIS, Fraser Health’s PH information system. Descriptive analyses were generated using Microsoft Excel (2020) software. The crude attack rate for secondary cases is the number of confirmed cases over the number of susceptible persons (i.e. close contacts who were not employed on the farm). Shared contacts were counted once.

## Interventions

### Farm 1

The provincial One Health Committee, detailed in **Table 1**, launched a tandem response to the Farm 1 outbreak. Public Health conducted an environmental health and occupational risk assessment on site, tested all workers and reviewed biosecurity practises. Simultaneously, MAFF placed Farm 1 under quarantine with restrictions on the transportation of animals, products, goods and people in and out of the site. Farm activities were restricted to those necessary for animal welfare; activities outside of this scope needed MAFF approval. An animal outbreak investigation identified no transmission between Farm 1 and other BC mink farms.

**Table 1:** Role of mink outbreak management working group

Name of outbreak management working group	Roles
Fraser Health Authority	Medical Health Officer—Clinically directed case and contact management, responsible for human outbreak control, enacted Public Health Orders Environmental Health Officer—Completed environmental health inspections, provided review of COVID-19 safety plans, retrieved mink mortalities from farms Communicable Disease Nurse Coordinator—Oversaw Cluster Investigators, provided clinical support to team, oversaw logistics of outbreak management Cluster Investigator—Reviewed cases that are employed at farms, input laboratory results, followed up with farms regarding issues related to vaccinations/testing, completed clinical assessment of farm cases Analyst—Monitored and processed laboratory data, summarized and analyzed epidemiology
BC Centre for Disease Control	Physician epidemiologist—Provided leadership in supporting One Health group, pulled together scientific literature, connected national stakeholders and international organizations Public health veterinarian—Provided expertise on the intersection of animal and human health, connected to federal advisory working groups Epidemiologist—Designed and implemented mink farmworker surveillance system, provided surveillance reports, liaised with MAFF veterinarian-epidemiologist Laboratory staff—Provided laboratory services including processing of weekly real-time polymerase chain reaction SARS-CoV-2 tests and completion of genomic sequencing of human cases, provided interpretation of genomic sequencing data Coordinated transportation of animal samples to the National Microbiology Laboratory for processing and sequencing
Ministry of Agriculture, Food and Fisheries	Responsible for health and well-being of animals, outbreak management in agricultural settings; performed testing of animals, provided guidance on risk reduction procedures
WorkSafeBC	Regulated safety of farmworkers, supported development of protocols and standards for occupational safety on farms
Ministry of Forests, Lands, Natural Resource Operations and Rural Development	Supported wildlife surveillance surrounding farms
Canadian Food Inspection Agency	Provided expert consultation and scientific advice
Ministry of the Environment	Regulated environmental discharges, including manure

Abbreviations: BC, British Columbia; COVID-19, coronavirus disease 2019; MAFF, Ministry of Agriculture, Food and Fisheries; SARS-CoV-2, severe acute respiratory syndrome coronavirus 2

The One Health Committee provided instructions for animal care, including minimizing interaction length and frequency, limiting care to asymptomatic individuals, and enhanced hand hygiene. Enhanced biosecurity measures for mink care included the use of full personal protective equipment (i.e. N95 masks, disposable Tyvex suit, long rubber gloves, rubber boots), the establishment of a quarantine zone for donning and doffing personal protective equipment, and sanitation procedures for soiled boots and gloves. Surgical masks were sufficient when not in close proximity to mink.

Three recovered farmworkers were permitted to euthanize the herd for the purposes of pelt production December 16–24 2020, as pelting would remove infected mink and reduce risk of further transmission. Breeding stock was retained. All euthanized mink were stored in the farm freezer for later processing. Farmworkers involved in pelting were released from isolation 14 days after the last mink was euthanized. Following pelting, no SARS-CoV-2 was detected in the herd.

### Farm 3

While the response to the Farm 3 outbreak had many similarities to Farm 1, the most significant difference in the two responses was the presence of industry-wide, preventative measures and surveillance infrastructure that was established from mid-December 2020 to April 2021. These measures included the creation of a weekly voluntary human surveillance system to detect asymptomatic or pre-symptomatic cases among farmworkers, as well as public health mandates to ensure weekly testing of mink mortalities, enhanced biosecurity measures (as described for Farm 1) and farmworker vaccination ((9)).

In addition to the above measures, PH required all Farm 3 workers exposed to the mink herd to follow a work-home quarantine May 11–June 9, 2021. The voluntary farmworker screening was increased to 2–3 times a week. Concurrent environmental health inspections found an acceptable compliance with the newly established provincial-level biosecurity requirements.

## Results

### Farm 1 outbreak

**Figure 1:**
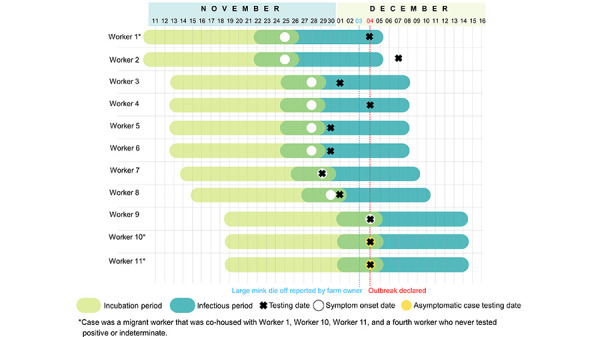
Gantt chart of confirmed and epidemiologically linked cases among mink farmworkers on Farm 1 in British Columbia, Canada

There were 11 cases among 12 farmworkers (8 confirmed cases; 3 epidemiologically linked cases) associated with the Farm 1 outbreak (**Table 2**). Cases had tightly clustered symptom onset November 25–December 4, 2020 (**Figure 1**). A large mink die-off followed the symptom onset of the 2 index cases by 8 days. Notably, 2 of 4 co-housed migrant workers were asymptomatic with high Ct values, suggesting remote infection and potentially earlier onset than reported to PH.

**Table 2:** Demographics and symptoms of confirmed and epidemiologically linked cases associated with mink farm COVID-19 outbreaks December 2020–October 31, 2021

Case demographics and symptoms	Overall		Farm 1		Farm 3	
	n	%	n	%	n	%
Number of cases	17	17	11	11	6	6
Case type						
Confirmed case	14	82.4	8	72.7	6	100
Epidemiologically linked case	3	17.6	3	27.3	0	0
Age group (years)						
20–39	7	41.2	5	45.5	2	33.3
40–79	10	58.8	6	54.5	4	66.7
Vaccination status						
No vaccination	11	64.7	11	100	0	0
Within 14 days of first dose	1	5.9	0	0	1	16.7
Two valid doses	5	29.4	0	0	5	83.3
Symptoms						
Asymptomatic	5	29.4	2	18.2	3	50.0
Chills	3	17.6	3	27.3	0	0
Fever	3	17.6	2	18.2	1	16.7
Runny nose	3	17.6	1	9.1	2	33.3
Sore throat	3	17.6	2	18.2	1	16.7
Cough	2	11.8	2	18.2	0	0
Fatigue	2	11.8	2	18.2	0	0
Myalgia	2	11.8	2	18.2	0	0
Nasal congestion	2	11.8	2	18.2	0	0

Abbreviation: COVID-19, coronavirus disease 2019

Environmental health inspection on December 5, 2020, identified weak infection control practices. Relevant findings included the use of cloth masks, a single handwashing station with a reusable towel and no log of entrance screening questions or cleaning, which may have facilitated mink-to-human transmission.

**Figure 2:**
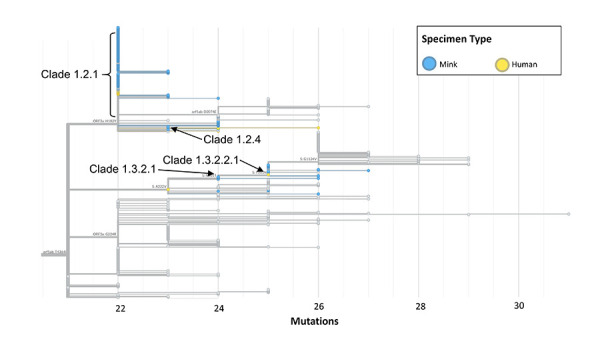
Farm 1 and a subset of community cases^a^ of SARS-CoV-2 identified in British Columbia, Canada, November 10, 2020–December 19, 2020 Abbreviation: SARS-CoV-2, severe acute respiratory syndrome coronavirus 2 ^a^ This tree contains only community cases classified as belonging to the SARS-CoV-2 PANGOLIN AW.1. There were additional lineages circulating at this time in British Columbia; however, these were not considered genetically related to Farm 1 cases and were subsequently excluded from this custom phylogenetic tree. The corresponding epidemic curve of confirmed SARS-CoV-2-positive mink and human samples is shown in the top right-hand corner. The index farmworker case with high quality sequencing data and onset on November 25, 2020 clustered into Clade 1.3.2.2.1. A household contact of the index case with low quality sequencing data clustered with four farmworker samples in Clade 1.2.1

Phylogenetic analysis for Farm 1 included 8/11 farmworkers, 6 close contacts and 151 mink samples that generated high-quality sequence data. All Farm 1 samples clustered within 1 distinct genetic clade (Clade 1) within the AW.1 lineage, a lineage circulating locally in October 2020 (**Figure 2**). Four farmworker samples and a sequenced household contact of an index case were genetically identical or nearly identical to one another and mink samples (Clade 1.2.1). The other sequenced index case clustered on a divergent branch of the tree (Clade 1.3.2.2.1). Mink isolates acted as genetic intermediaries between human sequences (Clade 1.2.4 and 1.3.2.1). Notably, community cases related to Clade 1.3.2.2.1 with onset after December 3, 2020, included clusters among vulnerable populations such as long-term care. Clades 1.3.2.2.1 and 1.2.1 were not detected through routine WGS surveillance in the community prior to the outbreak.

### Farm 3 outbreak

**Figure 3:**
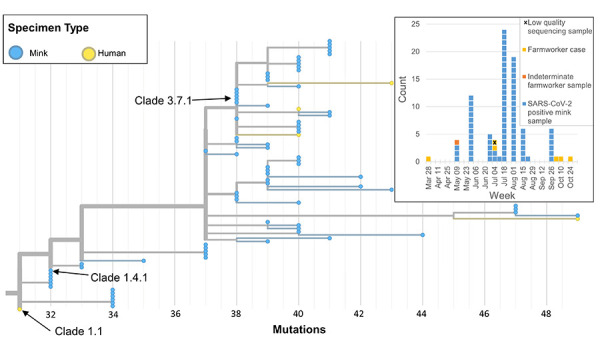
Farm 3 human and mink samples of SARS-CoV-2 identified in British Columbia, Canada, April 2, 2021–October 31, 2021 Abbreviation: SARS-CoV-2, severe acute respiratory syndrome coronavirus 2 Note: Corresponding epidemic curve of confirmed SARS-CoV-2-positive mink and positive and indeterminate farmworker samples is shown in the top right-hand corner. The first farmworker case with onset on April 2, 2021 was clustered into Clade 1.1

The epidemic curve of the Farm 3 outbreak resembles one expected from an intermittent source, spanned from April–October 2021, and had fewer human cases compared to Farm 1 (six confirmed cases) despite a similar workforce size (Table 2, **Figure 3**). Other than the index case, new human cases were associated with high levels of mink-human contact. The two confirmed cases with onset in July were associated with a personal protective equipment breach reported during a heat event in June 2021; while the three cases in October had onset after a period of intense animal relocation. When compared to Farm 1, the outbreak on Farm 3 had a greater proportion of asymptomatic cases (Farm 3=50.0%; Farm 1=18.2%, Table 2) and lower attack rates among close contacts (Farm 3=12.5%; Farm 1=29.4%).

Five of six human samples and 79 mink samples generated high-quality sequence data and were included in the phylogenetic analysis (Figure 3). Human and mink samples clustered tightly together on trees that diverged from the B.1.618 lineage. Mink sequences were genetically intermediate between human sequences (Clade 1.4.1 and Clade 3.7.1) and no human samples shared the same clade. While the B.1.618 lineage was circulating in the region at the time of the first human case (Clade 1.1), a more than 80% sequencing coverage of community cases during the time of subsequent human cases on Farm 3 detected no background community circulation of this strain. No community transmission of the Farm 3 strains was detected.

## Discussion

This report summarizes two COVID-19 outbreaks among mink farmers and their livestock in Canada. On both farms, symptoms were present in staff before detection in the minks, viral sequences from mink and human-derived isolates were closely related, and human cases predominantly arose during periods of higher human-mink contact (i.e. harvesting and animal relocation). These findings point to a likely anthropogenic introduction of SARS-CoV-2 into farmed mink by farm staff, viral evolution in the mink host and then reintroduction into human hosts. Variations in the length and transmission patterns observed between outbreaks may be attributable to the different PH measures in place at each outbreak’s onset.

The rapid transmission observed on Farm 1 may be credited to late detection, the absence of natural or vaccine-mediated immunity among farmworkers and mink and ineffective biosecurity control measures. Factors resulting in late outbreak detection included self-initiated community testing and early spread among the co-housed migrant workforce, a population well recognized for limited access to health services ((15,16)). Alternatively, the short course of the outbreak can be attributed to the exhaustion of the susceptible farmworker population (91.6% attack rate) and pelting of the mink herd eliminating the outbreak’s source.

The late detection of the Farm 1 outbreak makes it difficult to establish the date of introduction onto the farm and chain of transmission, which could plausibly have begun weeks earlier than the index case. While it is challenging to establish directionality of transmission for all cases, the WGS analysis strongly suggests two human cases acquired SARS-CoV-2 from mink. Mink-to-human transmission is further supported by the rapid genetic divergence observed on Farm 1, which is beyond the approximate one mutation per two weeks expected through human-to-human transmission alone ((12)).

The absence of related co-circulating community strains, dispersal of human cases in time, epidemiology and WGS pointed to multiple transmissions from mink to fully vaccinated humans over seven months at Farm 3. Although other outbreak reports have suggested outbreaks in farmed mink can run their course quickly ((2,6)), Farm 3’s outbreak timeline demonstrated that a herd outbreak can persist for months and function as an intermittent source of SARS-CoV-2. This complements existing evidence that mink can function as long-standing reservoirs of SARS-CoV-2 ((6,17)). While vaccination and enhanced biosecurity practises were able to reduce transmission risk, as displayed by the high proportion of asymptomatic cases (Farm 1=18.2%; Farm 3=50.0%) and month-long periods between farmworker cases in Farm 3 as compared to Farm 1, they were unable to eliminate mink-to-human transmission from an established mink reservoir. Research on farm-related factors contributing to prolonged infection in mink is needed to inform future prevention efforts.

The absence of spillover into the community stemming from Farm 3 illustrates the potential importance of provincial policies enacted in BC that led to the development of early outbreak detection systems, enhanced biosecurity measures and early vaccination of mink farmers and their households ((9)). Specifically, early detection of cases through biweekly worker surveillance and vaccinations may explain the absence of farmworker-to-farmworker transmission and the low attack rate among farmworkers’ close contacts on Farm 3 compared to Farm 1 (Farm 3=12.5%; Farm 1=29.4%). Simultaneously, the spillover of SARS-CoV-2 identified at Farm 1 into high-risk populations in the community, similar to previous reports in Denmark ((4)) and the Netherlands ((6)), illustrates the risk of late detection of infection in the mink farm setting. Alternatively, this difference may reflect the Farm 3 variant’s introduction into a highly vaccinated population at a time of high community prevalence of the Delta variant, the dominant lineage from July 4, 2021, onwards ((18,19)).

The strengths of this outbreak investigation included the adoption of a One Health approach that integrated multiple agencies to respond to a pathogen with demonstrated capacity for human spillovers. Comprehensive, frequent testing of staff during outbreaks makes the likelihood of undetected human intermediary cases remote.

### Limitations

There are several limitations to this report. Data on symptoms and close contacts were self-reported and vulnerable to social desirability and recall bias. Due to concerns about economic, logistical and reputational impacts, farmworkers and owners may have been hesitant to report a large number of contacts. It is difficult to ascertain a causal relationship between individual initiatives and their effects on controlling each outbreak given the application of multiple interventions. Despite these limitations, this report adds to the literature of the emerging threat of SARS-CoV-2 in mink reservoirs and describes the actions that led to the interruption of the chain of transmission of mink variants in the largest health authority in British Columbia.

## Conclusion

These outbreaks provide additional evidence of zoonotic transmission of SARS-CoV-2 from mink to humans and the potential for subsequent community spread. The second outbreak at Farm 3 demonstrated that the risk of human acquisition can persist for months during longer outbreaks in mink herds. Biosecurity requirements, staff vaccination and an ongoing surveillance system contributed to reducing the spillover of mink variants into the general community; however, these measures were unable to eliminate the risk of mink-to-human transmission during persistent herd infections. These outcomes provide evidence for other jurisdictions of the importance of active surveillance to support timely response to SARS-CoV-2 in these high-risk settings. A One Health approach is needed to successfully respond to outbreaks involving humans and animals, as experts in various fields must collaborate to limit disease spread.
